# Identification of robust and interpretable brain signatures of autism and clinical symptom severity using a dynamic time-series deep neural network

**DOI:** 10.1192/j.eurpsy.2021.397

**Published:** 2021-08-13

**Authors:** K. Supekar, S. Ryali, R. Yuan, D. Kumar, C. De Los Angeles, V. Menon

**Affiliations:** Psychiatry And Behavioral Sciences, Stanford University, Stanford, United States of America

**Keywords:** autism, biomarkers, brain dynamics, fMRI

## Abstract

**Introduction:**

Autism spectrum disorder (ASD) is among the most common and pervasive neurodevelopmental disorders. Yet, despite decades of research, the neurobiology of ASD is still poorly understood, as inconsistent findings preclude the identification of robust and interpretable neurobiological markers and predictors of clinical symptoms.

**Objectives:**

Identify robust and interpretable dynamic brain markers that distinguish children with ASD from typically-developing (TD) children and predict clinical symptom severity.

**Methods:**

We leverage multiple functional brain imaging cohorts (ABIDE, Stanford; N = 1004) and exciting recent advances in explainable artificial intelligence (xAI), to develop a novel multivariate time series deep neural network model that extracts informative brain dynamics features that accurately distinguish between ASD and TD children, and predict clinical symptom severity.

**Results:**

Our model achieved consistently high classification accuracies in cross-validation analysis of data from the ABIDE cohort. Crucially, despite the differences in symptom profiles, age, and data acquisition protocols, our model also accurately classified data from an independent Stanford cohort without additional training. xAI analyses revealed that brain features associated with the default mode network, and the human voice/face processing and communication systems, most clearly distinguished ASD from TD children in both cohorts. Furthermore, the posterior cingulate cortex emerged as robust predictor of the severity of social and communication deficits in ASD in both cohorts.

**Conclusions:**

Our findings, replicated across two independent cohorts, reveal robust and neurobiologically interpretable brain features that detect ASD and predict core phenotypic features of ASD, and have the potential to transform our understanding of the etiology and treatment of the disorder.

**Disclosure:**

No significant relationships.

